# Diverse Strategies for Vertical Symbiont Transmission among Subsocial Stinkbugs

**DOI:** 10.1371/journal.pone.0065081

**Published:** 2013-05-31

**Authors:** Takahiro Hosokawa, Mantaro Hironaka, Koichi Inadomi, Hiromi Mukai, Naruo Nikoh, Takema Fukatsu

**Affiliations:** 1 National Institute of Advanced Industrial Science and Technology (AIST), Tsukuba, Japan; 2 Department of Biology, Faculty of Medicine, Hamamatsu University School of Medicine, Hamamatsu, Japan; 3 Department of Applied Biological Sciences, Faculty of Agriculture, Saga University, Saga, Japan; 4 The United Graduate School of Agricultural Sciences, Kagoshima University, Kagoshima, Japan; 5 Department of Liberal Arts, The Open University of Japan, Chiba, Japan; International Atomic Energy Agency, Austria

## Abstract

Sociality may affect symbiosis and *vice versa*. Many plant-sucking stinkbugs harbor mutualistic bacterial symbionts in the midgut. In the superfamily Pentatomoidea, adult females excrete symbiont-containing materials from the anus, which their offspring ingest orally and establish vertical symbiont transmission. In many stinkbug families whose members are mostly non-social, females excrete symbiont-containing materials onto/beside eggs upon oviposition. However, exceptional cases have been reported from two subsocial species representing the closely related families Cydnidae and Parastrachiidae, wherein females remain nearby eggs for maternal care after oviposition, and provide their offspring with symbiont-containing secretions at later stages, either just before or after hatching. These observations suggested that sociality of the host stinkbugs may be correlated with their symbiont transmission strategies. However, we found that cydnid stinkbugs of the genus *Adomerus*, which are associated with gammaproteobacterial gut symbionts and exhibit elaborate maternal care over their offspring, smear symbiont-containing secretions onto eggs upon oviposition as many non-social stinkbugs do. Surface sterilization of the eggs resulted in aposymbiotic insects of slower growth, smaller size and abnormal body coloration, indicating vertical symbiont transmission via egg surface contamination and presumable beneficial nature of the symbiosis. The *Adomerus* symbionts exhibited AT-biased nucleotide compositions, accelerated molecular evolutionary rates and reduced genome size, while these degenerative genomic traits were less severe than those in the symbiont of a subsocial parastrachiid. These results suggest that not only sociality but also other ecological and evolutionary aspects of the host stinkbugs, including the host-symbiont co-evolutionary history, may have substantially affected their symbiont transmission strategies. (250 words).

## Introduction

Diverse insects are associated with symbiotic microorganisms of mutualistic, commensalistic or parasitic nature [1], [2]. In particular, vertical transmission is among the most pivotal processes underpinning mutualistic host-symbiont associations. For the symbiont side, failure in vertical transmission may be almost equivalent to death because, in general, the symbionts are obligatorily dependent on the hosts. For the host side, failure in symbiont transmission may result in significant fitness reduction, often leading to mortality and/or sterility. Under the selection pressures acting on both partners, it is expected that some mechanisms for ensuring vertical symbiont transmission should have evolved, which may be molecular, cellular, physiological, morphological, developmental and/or behavioral ones [1], [3]–[10].

The majority of plant-sucking stinkbugs of the superfamily Pentatomoidea harbor mutualistic bacterial symbionts in a posterior region of the midgut, where a number of crypts develop and their lumen harbor specific symbiotic bacteria [1], [7]. In these stinkbugs, adult females excrete symbiont-containing materials from the anus, which their offspring orally ingest and establish vertical symbiont transmission. In many stinkbug families whose members are mostly non-social, adult females excrete symbiont-containing materials upon oviposition, which are either directly smeared onto the egg surface in such families as the Pentatomidae and the Scutelleridae, or encased in “symbiont capsules” and placed besides the eggs in the family Plataspidae, and newborn nymphs ingest the materials and acquire the symbionts when they hatch [1], [7], [11]–[18].

However, exceptional cases have been reported from *Cydnus atterimus* ( = *Brachypelta atterima*) and *Parastrachia japonensis,* which belong to the closely related families Cydnidae and Parastrachiidae, respectively [19]. In these subsocial stinkbugs, adult females lay eggs within an underground nest and remain nearby their offspring for maternal care, which entails defense against natural enemies and food provisioning [20], [21]. Distinct from non-social stinkbugs, adult females of these subsocial stinkbugs excrete symbiont-containing fluid from the anus not upon oviposition but at later stages: after egg hatching in *C. atterimus* [20] and just before egg hatching in *P. japonensis* [9]. These observations suggest that sociality of the host stinkbugs may be strongly correlated with the timing and the strategy of vertical symbiont transmission. Microbiological characterization of the symbiont of *P. japonensis*, designated as “*Candidatus* Benitsuchiphilus tojoi”, estimated the genome size of the symbiont as small as 0.85 Mb, suggesting that the host-symbiont association has been stably maintained over evolutionary time [22].

**Figure 1:**
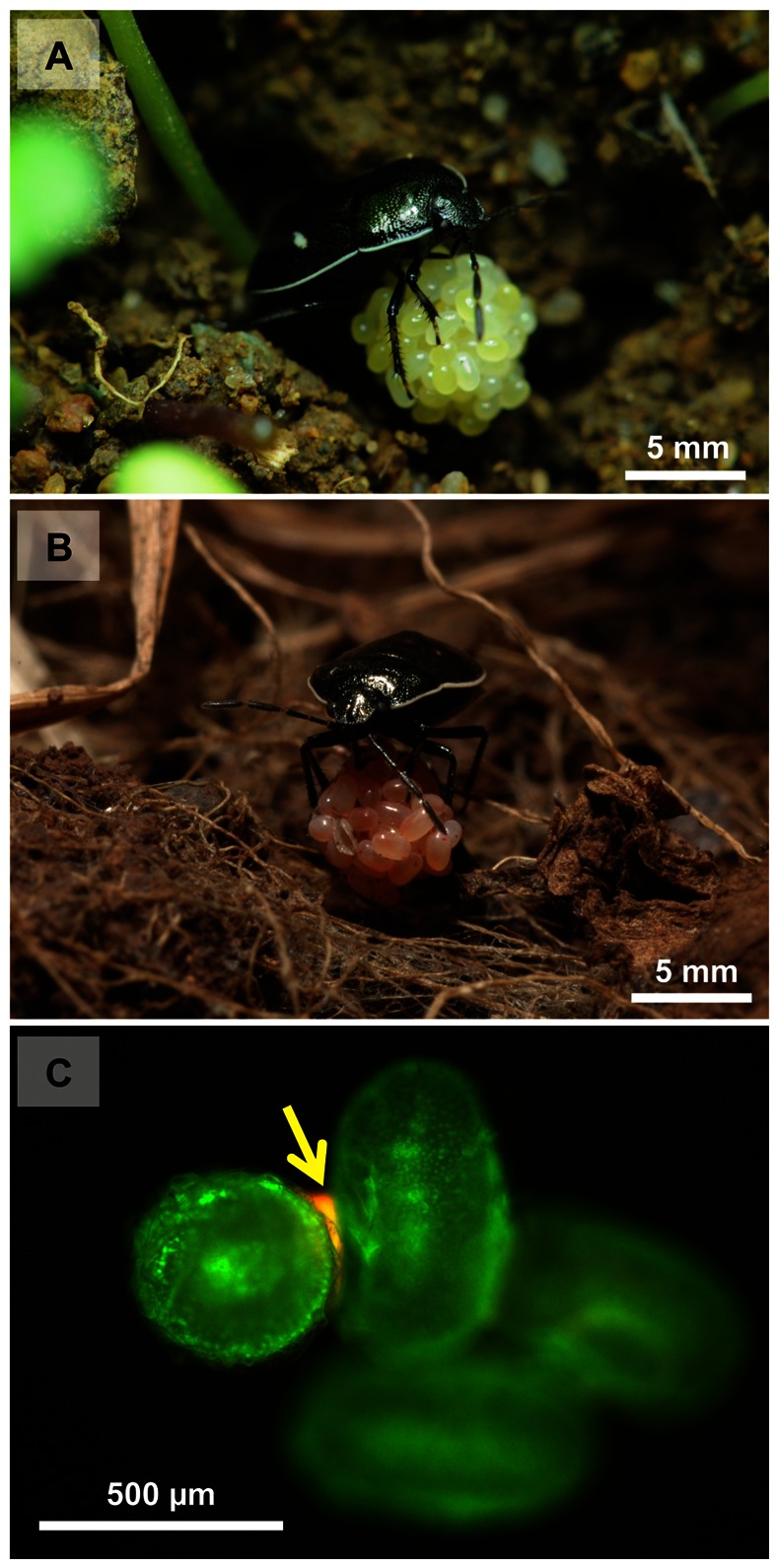
Egg masses of *Adomerus* stinkbugs. (A) and (B) An adult female of *A. triguttulus* and *A. rotundus* keeping an egg mass in the nest, respectively. (C) Visualization of the symbiont localization on freshly-laid eggs of *A. triguttulus* by whole-mount *in situ* hybridization. Red and green signals represent the symbiont 16S rRNA and autofluorescence of the egg shell, respectively. An arrow indicates the symbiont signals between the eggs.

Subsocial cydnid stinkbugs of the genus *Adomerus* are members of the subfamily Sehirinae, which is distinct from the subfamily Cydninae embracing *C. atterimus*. Adult females of *Adomerus triguttulus* and *A. rotundus* lay a ball-shaped egg mass within an underground nest (Fig. 1A and B), where they perform complex maternal behaviors including egg guarding, production of trophic eggs, protection of nymphs against enemies, and progressive provisioning of food plant nutlets [23]–[25]. Specific gammaproteobacterial symbionts were identified in the midgut crypts based on 16S rRNA gene sequences [26], but biological and genomic aspects of the *Adomerus* gut symbiosis have been poorly understood.

In this study, we investigated vertical transmission processes, fitness effects and genomic features of the symbionts of *Adomerus* stinkbugs, and found that, unexpectedly, despite their sociality, these stinkbugs smear symbiont-containing secretions onto the egg surface during oviposition, as many non-social stinkbugs do.

## Materials and Methods

### Insects

Adult insects of *A. triguttulus* and *A. rotundus* were collected in 2007 from their host plants, the purple deadnettle *Lamium purpureum* at Tsukuba, Japan, and the henbit deadnettle *Lamium amplexicaule* at Karatsu, Japan, respectively. No specific permits were required for the field studies. The locations are not privately-owned or protected in any way. The field studies did not involve endangered or protected species.

The insects were kept in 1.5-L plastic containers with moistened paper towel and sufficient nutlets of the host plants as food. The containers were checked daily. When we found females guarding an egg mass, each set of a female and an egg mass was transferred to a 0.1-L container and individually maintained with moistened paper towel and sufficient nutlets until experiments. These insects were kept at 20°C for *A. triguttulus* and 27°C for *A. rotundus* under a long-day regimen of 16 h light and 8 h dark.

### Symbiont Detection

Egg masses collected within 24 h after oviposition (n = 9 for *A. triguttulus* and n = 8 for *A. rotundus*) were individually subjected to DNA extraction using the QIAamp DNA Mini Kit (QIAGEN). The DNA samples were subjected to diagnostic PCR using the primers 16SA1 (5′-AGA GTT TGA TCM TGG CTC AG-3′) and MTBS16S (5′-GGC TAA TAA CCG TGT CGT-3′) for 16S rRNA gene of the symbiont of *A. triguttulus*, and 16SA1 and FTBS16S (5′-GGT GAT ATG GAT AAT ACC CAT-3′) for 16S rRNA gene of the symbiont of *A. rotundus*, under the temperature profile of 95°C for 10 min followed by 35 cycles consisting of 95°C for 30 sec, 55°C for 1 min, and 72°C for 1 min. Normal egg masses of *A. triguttulus* and *A. rotundus* consist of more than 70 eggs [23], [27]. However, we were occasionally able to collect small incomplete egg masses in the midway of formation when we found adult females laying eggs. Such small egg masses consisting of less than 10 eggs (n = 3 for *A. triguttulus* and n = 2 for *A. rotundus*) were also individually subjected to DNA extraction and diagnostic PCR detection of the symbionts. Several egg masses of *A. triguttulus* were subjected to symbiont visualization by fluorescence *in situ* hybridization with a fluorochrome-labeled oligonucleotide probe specifically targeting 16S rRNA of the symbiont as described [26] and observed under an epifluorescence microscope (Axiophot; Carl Zeiss). Several egg masses of *A. rotundus* were subjected to surface observation with a scanning electron microscope (S-4800; Hitachi).

### Detection of Vertical Symbiont Transmission

Adult females that had freshly laid an egg mass within 24 h were collected, each of the female-offspring sets (n = 3 for *A. triguttulus* and n = 3 for *A. rotundus*) was transferred to a plastic container, the adult females were removed, and each of the isolated egg masses was kept in the container and inspected daily. Approximately 24 h after hatching, ten nymphs were randomly picked from each of the broods and individually subjected to DNA extraction and diagnostic PCR detection of the symbionts.

### Behavioral Observation

Under the rearing conditions, the eggs hatched 10–11 days after oviposition for *A. triguttulus* [23] and 6–7 days after oviposition for *A. rotundus* [25]. To observe behavioral events associated with the egg hatching, we prepared female-offspring sets whose eggs were estimated to hatch within 24 h (n = 4 for *A. triguttulus* and n = 7 for *A. rotundus*), and they were continuously monitored either by direct observation or by using a video camera (HDR-XR500V, SONY).

### Egg Surface Sterilization and Fitness Measurement

We prepared female-offspring sets whose egg masses were estimated to hatch within 24 h. After the females were removed, the egg masses were randomly assigned to either of the following experimental groups: the control group, in which the egg masses were soaked in water for 40 min (n = 14 for *A. triguttulus* and n = 5 for *A. rotundus*), or the sterilized group, in which the egg masses were soaked in 70% ethanol for 10 min and subsequently in 4% formaldehyde for 30 min, and finally rinsed with 70% ethanol (n = 10 for *A. triguttulus* and n = 5 for *A. rotundus*). After air-drying, these egg masses were returned to the original containers and allowed to hatch without mothers. Approximately 24 h after hatching, ten nymphs were randomly selected from each of the broods, transferred to a new rearing container, and fed with sufficient nutlets until all nymphs reached adulthood or died. These insects were examined for adult emergence rate, nymphal period and adult dry weight, and finally subjected to DNA extraction and diagnostic PCR detection of the symbionts.

### Molecular Evolutionary Analysis

From the midgut DNA samples of *A. triguttulus* and *A. rotundus*, *gyrB* and *groEL* genes of the symbionts were amplified by PCR, cloned and sequenced as described [22]. Sequences of 16S rRNA gene of the symbionts of *A. triguttulus* and *A. rotundus* were previously reported [26], so were sequences of 16S rRNA, *gyrB* and *groEL* genes of the symbiont of *P. japonensis* [22]. In addition, we determined *gyrB* and *groEL* gene sequences of the gut symbionts of cydnid stinkbugs *Adrisa magna* and *Macroscytus japonensis*, while their 16S rRNA gene sequences were previously reported [26]. These gene sequences were subjected to relative rate tests using the program RRTree [28] based on genetic distances estimated by Kimura’s two-parameter model [29].

### Symbiont Genome Sizing

The posterior midgut section with symbiont-harboring crypts was dissected from three adult females of *A. rotundus*, homogenized in phosphate-buffered saline containing 10 mM EDTA, and filtered through a 20 µm nylon net filter. The symbiont cells obtained were embedded in 1% low melting point agarose, cut into plugs, and treated with proteinase K at 50°C overnight. After thorough washing, the symbiont DNA trapped within the plugs was digested with a restriction enzyme *I-CeuI*, and analyzed by pulsed-field gel electrophoresis using the CHEF Mapper XA (Bio-Rad).

### Statistical Analysis

Adult emergence rate was analyzed by generalized linear models with binomial or quasi-binomial error distribution [30]. Nymphal period and adult body weight were analyzed by generalized linear mixed models with Poisson and Gaussian error distributions, respectively, wherein the egg mass (brood) was treated as a random effect [31]. All the statistical analyses were conducted using the software R v.2.14.2. [32].

### Nucleotide Sequence Accession Numbers

The nucleotide sequences reported in this study have been deposited in the DDBJ/EMBL/GenBank databases under accession numbers AB781081 to AB781088.

## Results

### *Adomerus* Females Excrete Symbiont-containing Materials onto Eggs during Oviposition

**Figure 2:**
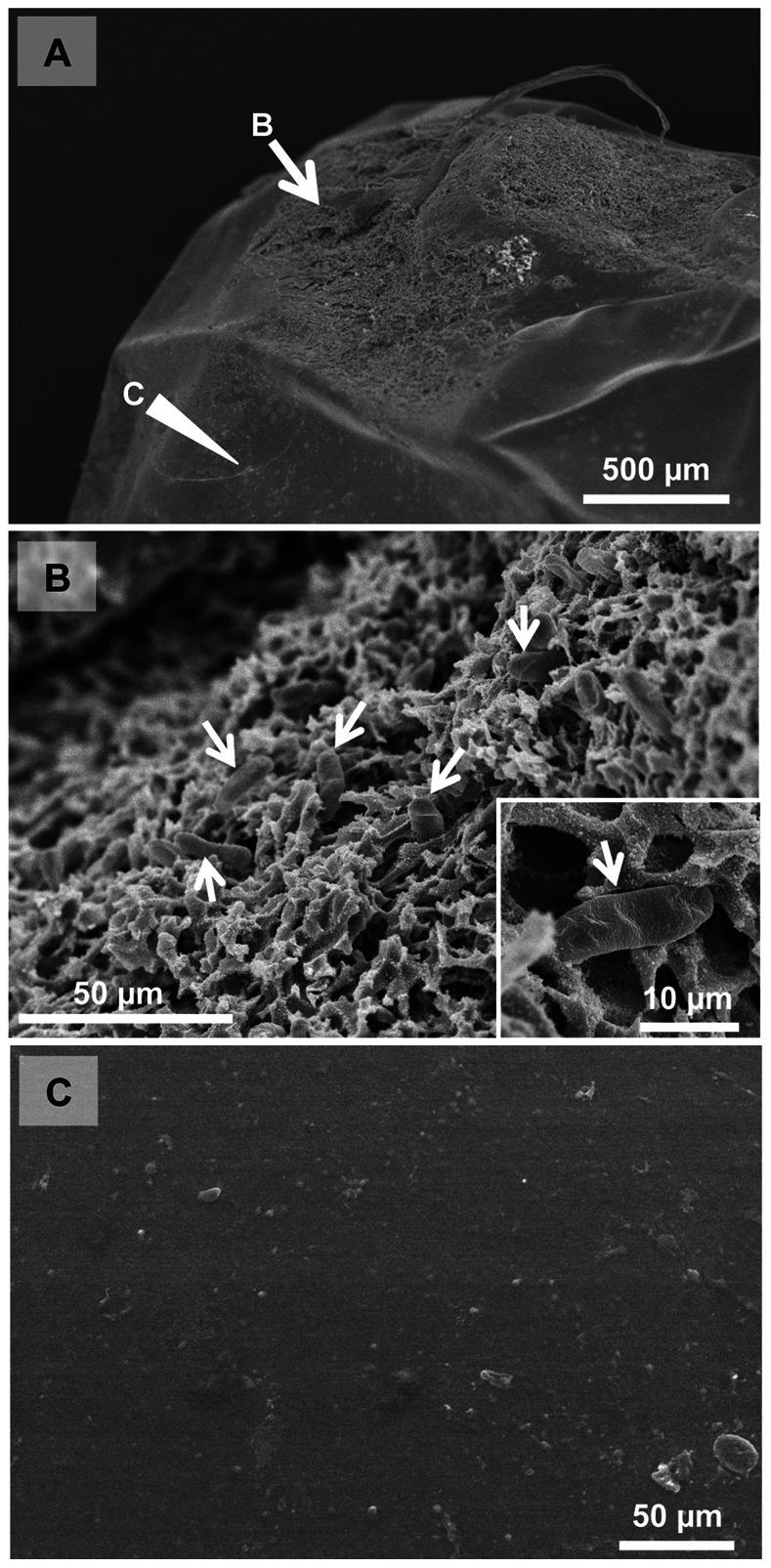
Scanning electron microscopy of the egg surface of *A.* *rotundus*. (A) A low magnification image. An arrow indicates the area that was attached to a neighboring egg by symbiont-containing secretion. An arrowhead indicates the area without the secretion. (B) An enlarged image of the area indicated by the arrow in (A). A number of symbiont cells (arrows) are buried in a layer of secretion on the egg surface. Inset shows a magnified image of a bacterial cell. (C) An enlarged image of the area indicated by the arrowhead in (A).

Diagnostic PCR revealed that all freshly-laid egg masses (n = 9 for *A. triguttulus* and n = 8 for *A. rotundus*) were symbiont positive. Fluorescence *in situ* hybridization detected the symbiont signals on the egg surface, where strong signals were observed between the eggs in association with gluing secretions (Fig. 1C). Scanning electron microscopy confirmed the presence of symbiont cells on the egg surface, which were buried in the gluing secretions (Fig. 2). When small egg masses in the midway of formation were subjected to diagnostic PCR, all of them (n = 3 for *A. triguttulus* and n = 2 for *A. rotundus*) were symbiont positive. These results indicate that *Adomerus* females excrete symbiont-containing materials onto the egg surface during oviposition.

### *Adomerus* Symbionts Smeared on the Egg Surface are Vertically Transmitted to Newborn Nymphs

When newborn nymphs from the orphan egg masses (n = 3 for *A. triguttulus* and n = 3 for *A. rotundus*), from which females had been experimentally removed just after oviposition, were subjected to diagnostic PCR, all the nymphs (in total n = 30 for *A. triguttulus* and n = 30 for *A. rotundus*) were symbiont positive. When insects from the surface-sterilized egg masses, whose surface had been treated with ethanol and formalin, were subjected to diagnostic PCR at the adult stage, only 2.5–3.7% of the insects were symbiont positive (2/54 insects for *A. triguttulus* and 1*/*40 insects for *A. rotundus*), whereas 100% of the insects from the control egg masses were symbiont positive (109/109 insects for *A. triguttulus* and 41/41 insects for *A. rotundus*). These results indicate that (i) presence of females upon egg hatching is not required for vertical symbiont transmission and (ii) symbiotic bacteria smeared on the egg surface contribute to vertical symbiont transmission.

### Behaviors of *Adomerus* Females and Nymphs around Egg Hatching

Continuous behavioral observations of *Adomerus* females and associated mature egg masses identified no recognizable maternal secretions before, during and after egg hatching. In *A. triguttulus*, the egg masses consist of both fertilized eggs and trophic eggs [23]. We observed that, just after egg hatching, newborn nymphs of *A. triguttulus* started to probe the surface of the egg mass, probably exploiting the trophic eggs and the symbiont-containing secretions. In *A. rotundus*, the egg masses consist mostly of fertilized eggs, and females start to lay trophic eggs just after egg hatching [25]. We observed that newborn nymphs of *A. rotundus* actively exploited the mother-provisioned trophic eggs. Hence, it seems likely that, in both *A. triguttulus* and *A. rotundus*, the symbionts are vertically transmitted just after egg hatching via nymphal exploitation of the symbiont-containing secretions smeared on the egg surface. In *A. rotundus*, meanwhile, the possibility of additional maternal provisioning of the symbiont after egg hatching together with the trophic eggs cannot be ruled out.

### Fitness Effects of Symbiont Elimination on *Adomerus* Stinkbugs

**Figure 3:**
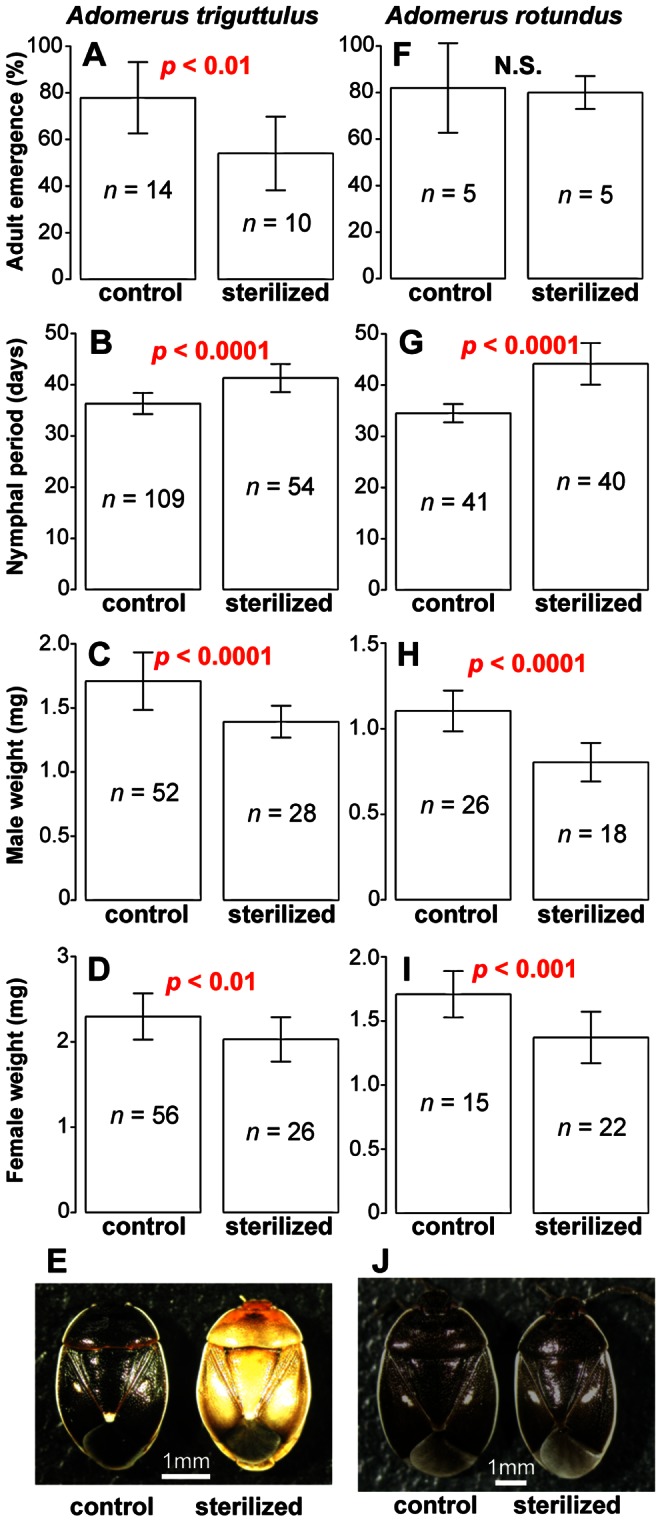
Effects of symbiont elimination on phenotypes of *Adomerus* stinkbugs. (A–E) *A. triguttulus*. (F–J) *A. rotundus*. (A, F) Adult emergence rate (%). (B, G) Nymphal period (days). (C, H) Dry body weight of adult males (mg). (D, I) Dry body weight of adult females (mg). (E, J) External appearance of newly emerged adult insects. Means and standard deviations are shown. Statistically significant differences between the control treatment and the sterilized treatment are highlighted in red.

Aposymbiotic insects of *A. triguttulu*s from the surface-sterilized egg masses exhibited lower adult emergence rate, longer nymphal period, smaller body size and abnormal pale coloration in comparison with symbiotic insects from the control egg masses (Fig. 3A–E). Similarly, aposymbiotic insects of *A. rotundus* from the surface-sterilized egg masses exhibited longer nymphal period and smaller body size in comparison with symbiotic insects from the control egg masses (Fig. 3F–J). These results suggest that the symbionts play important biological roles for normal growth and development of the *Adomerus* stinkbugs.

### Molecular Evolutionary Aspects of Gut Symbionts of *Adomerus* Species and Other Cydnid and Parastrachiid Stinkbugs

**Figure 4:**
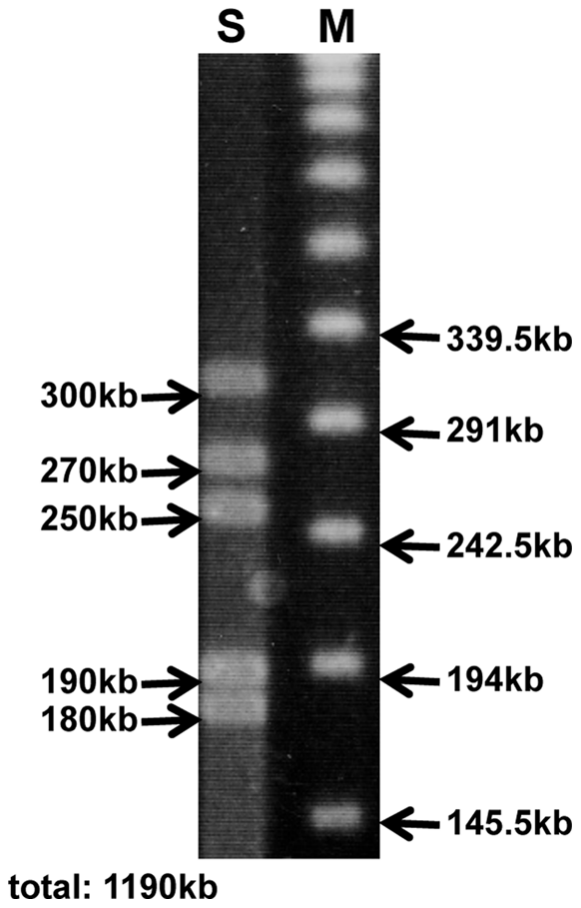
Genome size of the symbiont of *A.* *rotundus* estimated by pulsed-field gel electrophoresis. Lane S, symbiont genomic DNA digested with *I-CeuI*; lane M, DNA size markers.

Table 1 shows AT contents of 16S rRNA, *gyrB* and *groEL* genes of the gut symbionts of *Adomerus* species and other cydnid and parastrachiid stinkbugs, and also free-living gammaproteobacteria. In general, free-living gammaproteobacteria like *Escherichia coli* and *Salmonella enterica* exhibit around 45% AT values within these genes. The gut symbionts of *Adrisa magna* and *Macroscytus japonensis*, from which neither maternal care over offspring nor other social behaviors have been reported, exhibited AT values similar to *E. coli* and *S. enterica*, ranging from 44.4% to 46.2%. By contrast, the gut symbionts of *A. triguttulus* and *A. rotundus* showed remarkably higher AT values for *gyrB* and *groEL* genes (56.7%–58.3% and 54.1%–55.5%, respectively), although AT values for 16S rRNA gene were similar to those of free-living bacteria (45.7%–46.2%). Notably, the gut symbiont of *P. japonensis* exhibited the highest AT values, 51.0%, 70.5% and 65.0% for 16S rRNA, *gyrB* and *groEL* genes, respectively, which must reflect the degenerative evolution of the symbiont genome over evolutionary time [22]. Relative rate tests of the gene sequences revealed similar molecular evolutionary patterns. The gut symbionts of *A. triguttulus* and *A. rotundus* exhibited significantly higher molecular evolutionary rates in 16S rRNA, *gyrB* and *groEL* genes than the gut symbionts of *A. magna* and *M. japonensis* and also the free-living gammaproteobacteria, whereas the evolutionary rates were significantly lower than those of the gut symbiont of *P. japonensis* (Table 2). Figure S1 shows phylogenetic relationships between the symbionts of *A. triguttulus*, *A. rotundus*, and other cydnid stinkbugs together with related gammaproteobacteria based on the 16S rRNA, *gyrB* and *groEL* gene sequences. Pulsed-field gel electrophoresis of the gut symbiont of *A. rotundus* estimated its genome size as 1.2 Mb (Fig. 4), which is drastically smaller than the genomes of free-living gammaproteobacteria (ex. 4.6 Mb for *E. coli* and 4.9 Mb for *S. enterica*) [33], [34] but still considerably larger than the 0.85 Mb genome of the gut symbiont of *P. japonensis* [22].

**Table 1:** AT contents of symbiont genes.

Ecological information on host and symbiont	Symbiont and host species	16S rRNA [accession no.]	*gyrB* [accession no.]	*groEL* [accession no.]
	Symbiont of *Adomerus triguttulus* (Cydnidae)	46.2% (677/1,465) [AB703083]	58.3% (539/925) [AB781081]	54.1% (863/1,595) [AB781085]
Subsocial host stinkbugs with elaborate maternal care over offspring; vertical symbiont transmission from mothers to offspring confirmed	Symbiont of *Adomerus rotundus* (Cydnidae)	45.7% (669/1,464) [AB703086]	56.7% (523/922) [AB781082]	55.5% (885/1,595) [AB781086]
	Symbiont of *Parastrachia japonensis* (Parastrachiidae)	51.0% (763/1,497) [AB548050]	70.5% (650/922) [AB548057]	65.0% (1,037/1,595) [AB548064]
Neither sociality of host stinkbugs nor elaborate maternal care over offspring known; vertical symbiont transmission not examined	Symbiont of *Adrisa magna* (Cydnidae)	44.7% (654/1,464) [AB703082]	46.1% (425/922) [AB781083]	46.2% (738/1,598) [AB781087]
	Symbiont of *Macroscytus japonensis* (Cydnidae)	44.8% (656/1463) [AB703078]	44.4% (409/922) [AB781084]	46.0% (735/1,598) [AB781088]
Free-living gammaproteobacteria as control	*Escherichia coli*	45.2% (661/1,464) [AP009048]	44.4% (409/922) [AP009048]	47.0% (749/1,592) [AP009048]
	*Salmonella enterica*	45.2% (661/1,463) [U88545]	44.3% (408/922) [NC_003198]	45.4% (722/1,592) [U01039]

**Table 2:** Relative rate tests for comparing molecular evolutionary rates of 16S rRNA, *gyrB* and *groEL* genes of the symbionts.

Gene	Lineage 1	Lineage 2	Outgroup	K1^a^	K2^b^	K1– K2	K1/K2	*P* c
16S rRNA	Symbionts of *Adomerus triguttulus* 1 and *A. rotundus* 2	Symbiont of *Parastrachia japonensis* 3	*Vibrio cholerae* 8	0.034	0.063	−0.029	0.54	1.7×10^−3^
	Symbionts of *Adomerus triguttulus* 1 and *A. rotundus* 2	Symbionts of *Adrisa magna* 4 and *Macroscytus japonensis* 5	*Vibrio cholerae* 8	0.040	0.016	0.024	2.5	2.6×10^−4^
	Symbionts of *Adomerus triguttulus* 1 and *A. rotundus* 2	*Escherichia coli* 6 and *Salmonella enterica* 7	*Vibrio cholerae* 8	0.032	0.025	0.007	1.3	0.24
*gyrB*	Symbionts of *Adomerus triguttulus* 9 and *A. rotundus* 10	Symbiont of *Parastrachia japonensis* 11	*Vibrio cholerae* 15	0.061	0.134	−0.073	0.46	5.9×10^−4^
	Symbionts of *Adomerus triguttulus* 9 and *A. rotundus* 10	Symbionts of *Adrisa magna* 12 and *Macroscytus japonensis* 13	*Vibrio cholerae* 15	0.061	0.024	0.037	2.5	2.1×10^−3^
	Symbionts of *Adomerus triguttulus* 9 and *A. rotundus* 10	*Escherichia coli* 6 and *Salmonella enterica* 14	*Vibrio cholerae* 15	0.066	0.034	0.032	1.9	0.018
*groEL*	Symbionts of *Adomerus triguttulus* 16 and *A. rotundus* 17	Symbiont of *Parastrachia japonensis* 18	*Vibrio cholerae* 15	0.032	0.054	−0.022	0.59	0.032
	Symbionts of *Adomerus triguttulus* 16 and *A. rotundus* 17	Symbionts of *Adrisa magna* 19 and *Macroscytus japonensis* 20	*Vibrio cholerae* 15	0.049	0.015	0.034	3.3	1.8×10^−5^
	Symbionts of *Adomerus triguttulus* 16 and *A. rotundus* 17	*Escherichia coli* 6 and *Salmonella enterica* 21	*Vibrio cholerae* 15	0.049	0.016	0.033	3.1	4.5×10^−5^

Accession numbers of DNA sequences:

1 AB703083,

2 AB703086,

3 AB548050,

4 AB703082,

5 AB703078,

6 AP009048,

7 U88545,

8 X74694,

9 AB781081,

10 AB781082,

11 AB548057,

12 AB781083,

13 AB781084,

14 NC_003198,

15 NC_002505,

16 AB781085,

17 AB781086,

18 AB548064,

19 AB781087,

20 AB781088,

21 U01039.

a K1 is the estimated mean distance between lineage 1 and the last common ancestor of lineages 1 and 2.

b K2 is the estimated mean distance between lineage 2 and the last common ancestor of lineages 1 and 2.

c *P* values were generated using the program RRTree (28).

## Discussion

In the majority of non-social stinkbugs, their solitary lifestyle constrains the possible timing of symbiont transmission, because the opportunity of mother-offspring contact for vertical symbiont transmission is usually restricted to a short period around oviposition. By contrast, in subsocial stinkbugs like *C. atterimus* and *P. japonensis*, mother insects continuously stay with and take care of their offspring, which enables vertical symbiont transmission in a much broader time frame ranging from oviposition to nymphal stages. It has been argued that these sociality-related life history traits might have shaped their different strategies for vertical symbiont transmission [9]. In this study, however, we discovered that, even in subsocial *Adomerus* stinkbugs that exhibit elaborate social behaviors for taking care of their offspring, vertical symbiont transmission occurs via egg surface contamination during oviposition, as in many non-social stinkbugs. These results suggest that not only sociality but also other ecological and evolutionary aspects of the host stinkbugs may have substantially affected their symbiont transmission strategies. Hereafter, we discuss several ecological and evolutionary factors that may potentially be relevant to the different timing of vertical symbiont transmission.

In the majority of plant-sucking stinkbugs, eggs are laid on the surface of their host plants, and the symbiotic bacteria are either smeared on the egg surface or provided as symbiont capsules upon oviposition [1], [7], [11]–[18]. Therefore, the symbiotic bacteria are exposed to environmental stresses such as desiccation, UV irradiation and temperature fluctuation for several days or weeks until the eggs hatch and the newborn nymphs ingest them. In subsocial cydnid and parastrachiid stinkbugs, eggs are laid in an underground nest, where the environment is humid and rich in microorganisms and microarthropods. In particular, females of *P. japonensis* breed mainly in June, the humid and warm monsoon season in Japan [21], which may favor microbial contamination. Hence, it is conceivable, although speculative, that such environmental conditions may be fatal for the excreted symbiotic bacteria in the subsocial stinkbugs, and have facilitated the evolution of symbiont provisioning synchronous to egg hatching in *P. japonensis* [9]. On the other hand, females of *A. triguttulus* breed from mid April to early May, the cool spring season in Japan (Hosokawa et al. unpublished data), which may be relevant to the symbiont excretion upon oviposition and the symbiont survival outside the host body during the egg stage in *A. triguttulus*. In this context, it should be noted that some stinkbugs of the family Acanthosomatidae are known as subsocial, in which adult females lay an egg mass on the open plant surface, keep the eggs under their body, and defend the offspring against enemies [35]–[38], but they excrete symbiont-containing materials onto eggs upon oviposition [14], [39]. In future studies, more detailed ecological aspects of other subsocial stinkbugs, including *C. atterimus*, *A. rotundus and A. variegatus* [20], [25], [40], should be examined.

We experimentally showed that, in both *A. triguttulus* and *A. rotundus*, surface sterilization of the eggs results in successful elimination of the symbionts from the nymphs, and these aposymbiotic insects suffer attenuated fitness values and other phenotypic abnormalities (Fig. 3). These results strongly suggest that the symbionts play important biological roles for normal growth and development of the *Adomerus* stinkbugs. Although exact biological roles of the symbionts are unknown, it is conceivable that the symbionts may contribute to the host insects either via provisioning of nutritional elements or via metabolism of toxic compounds, as suggested for gut symbiotic bacteria of other stinkbugs [9], [11], [12], [14], [15], [17], [18], [20], [41]–[43]. Notably, the aposymbiosis-associated abnormalities look more severe in *A. triguttulus* than in *A. rotundus*, particularly in adult emergence rate (Fig. 3A and F) and adult body color (Fig. 3E and J). The differences suggest that *A. triguttulus* is more dependent on its symbiont than *A. rotundus* is, which may reflect different ecological and evolutionary aspects of the stinkbug species such as habitats, food plants, host-symbiont co-evolutionary history, or others. Meanwhile, the possibility cannot be excluded that the abnormalities observed in the aposymbiotic insects are actually attributable to the sterilization treatment rather than to the aposymbiosis. These issues should be addressed in future studies.

Among diverse insect-microbe symbiotic associations, symbiont genome reduction is correlated with host-symbiont co-evolution and interdependence. In general, free-living bacteria have the largest genomes of several megabases, facultative endosymbionts and endoparasites tend to exhibit moderately reduced genomes sometimes down to 1 Mb, and obligate mutualistic endosymbionts usually possess drastically reduced genomes smaller than 1 Mb and sometimes down to less than 0.2 Mb [44], [45]. Such reductive genome evolution, typically entailing AT-biased nucleotide compositions, accelerated molecular evolution and reduced genome size, is hypothesized to be the result of stable and nutrition-rich endosymbiotic environment and also the consequence of attenuated purifying selection due to small population size and strong bottleneck, which are associated with the endosymbiotic lifestyle of the vertically transmitted symbionts [44], [46], [47]. Recent studies have revealed that such reductive genome evolution is observed not only in endocellular symbiotic bacteria but also in extracellular symbiotic bacteria including the gut symbionts of stinkbugs [14], [22], [41], [48]. It is expected that, as a host-symbiont mutualistic association continues longer, the symbiont becomes more dependent on the life within the host, gradually loses genes needed for independent life, and, consequently, evolves the smaller genome. In *P. japonensis*, probably because of intimate host-symbiont association over evolutionary time, the genome size of the gut symbiont has become as small as 0.85 Mb [22]. The genome size is approaching the genome sizes of some obligate endocellular symbionts like *Blochmannia* of ants [49], [50], and may be too small to sustain the survival outside the host body for an extended period on the egg surface, which may constitute the reason why *P. japonensis* has evolved the fine-tuned behavioral mechanism for vertical symbiont transmission synchronous to egg hatching [9]. In *A. rotundus* (and probably also in *A. triguttulus*), by contrast, the genome size of the gut symbiont is about 1.2 Mb (Fig. 4), which is certainly reduced but equivalent to the genome sizes of many facultative endosymbionts and endoparasites [44], [47]. AT contents of the symbiont genes (Table 1) and molecular evolutionary rates (Table 2) exhibited similar degenerative evolutionary patterns. On the basis of these observations, although speculative, we hypothesize that (i) the evolutionary history of host-symbiont association in the *Adomerus* stinkbugs is younger than that in *P. japonensis*, (ii) the genome of the *Adomerus* symbiont is thus considerably larger than the genome of the *P. japonensis* symbiont, (iii) consequently, the *Adomerus* symbiont is able to survive outside the host body on the egg surface while the *P. japonensis* symbiont is not, (iv) these genomic difference between the *Adomerus* symbiont and the *P. japonensis* symbiont may underlie their different mechanisms of vertical symbiont transmission. In this context, these genomic features of the gut symbiont of the subsocial cydnid stinkbug *C. atterimus* are of interest and deserve future investigation.

In summary, we investigated vertical transmission, fitness consequences and genomic features of the gut symbionts of *A. triguttulus* and *A. rotundus*, and found that, unexpectedly, the symbionts are vertically transmitted via egg surface contamination upon oviposition, and, furthermore, the symbiont genome has experienced a moderate level of genome reduction. These findings suggest that not only sociality but also other ecological and evolutionary aspects of the host stinkbugs, including the host-symbiont co-evolutionary history, may have substantially affected and/or constrained their symbiont transmission strategies. Considering that symbiotic microorganisms ubiquitously play a variety of biological roles in diverse social insects such as termites, ants, honeybees, bark beetles and others [2], [51]–[54], relevance of our findings is not restricted to subsocial stinkbugs but may be reaching to other social insect systems intimately associated with microorganisms.

## Supporting Information

Figure S1 **Phylogenetic relationships between the symbionts of** ***A. triguttulus*** **,** ***A. rotundus*** **, and other cydnid stinkbugs.** Neighbor-joining trees based on (A) 1,428 aligned nucleotide sites of the 16S rRNA gene, (B) 305 amino acid sites of GyrB, and (C) 513 amino acid sites of GroEL. On each of the nodes, the bootstrap value of >50% is shown. In brackets are sequence accession numbers.
(TIF)
