# Supplementary figures and images for: Diverse Strategies for Vertical Symbiont Transmission among Subsocial Stinkbugs
Source: PLoS One. 2013 May 31;8(5):e65081. doi: 10.1371/journal.pone.0065081 (PMC3669201; doi:10.1371/journal.pone.0065081)

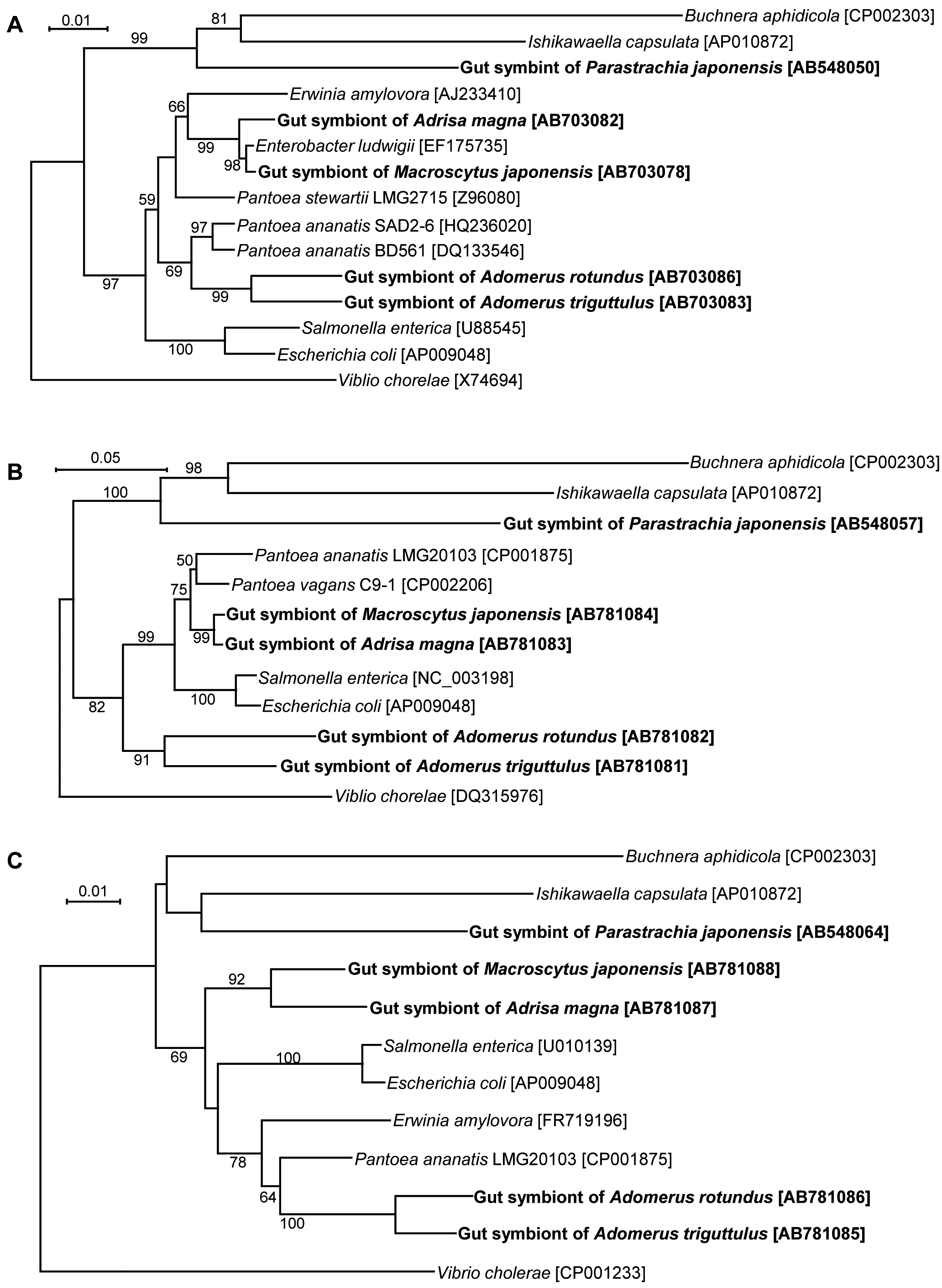

Supplement: Figure S1 — Phylogenetic relationships between the symbionts of A. triguttulus , A. rotundus , and other cydnid stinkbugs. Neighbor-joining trees based on (A) 1,428 aligned nucleotide sites of the 16S rRNA gene, (B) 305 amino acid sites of GyrB, and (C) 513 amino acid sites of GroEL. On each of the nodes, the bootstrap value of >50% is shown. In brackets are sequence accession numbers. (TIF) [file pone.0065081.s001.tif]
